# Development of a metabolic syndrome prediction model using smartphone-derived digital anthropometry

**DOI:** 10.1017/S000711452510576X

**Published:** 2026-01-28

**Authors:** Caleb F. Brandner, Grant M. Tinsley, Abby T. Compton, Sydney H. Swafford, Molly F. Johnson, Maria G. Kaylor, Hunter Haynes, Jon Stavres, Austin J. Graybeal

**Affiliations:** 1 Department of Health and Human Physiology, University of Iowa, Iowa City, IA, USA; 2 Department of Kinesiology and Sports Management, Texas Tech University, Lubbock, TX, USA; 3 School of Kinesiology and Nutrition, University of Southern Mississippi, Hattiesburg, MS, USA; 4 Department of Kinesiology, Nutrition, and Health, College of Education, Health, and Society, Miami University, Oxford, OH 45056, USA; 5 Department of Kinesiology, Harris College of Nursing and Health Sciences, Texas Christian University, Fort Worth, TX 76129, USA

**Keywords:** Mobile application, 3D scanning, Digital imaging, Cardiometabolic health, Smartphone

## Abstract

Integrating metabolic syndrome (MetS) screening procedures into routine care remains challenging. Traditional anthropometric and body composition assessments, while useful, have drawbacks that limit their application. However, automated anthropometrics produced from smartphone scanning applications may offer a solution. This study aimed to determine whether smartphone-derived anthropometrics could effectively predict both MetS and its severity. A total of 281 participants underwent a MetS screening assessment to determine fasting blood pressure, lipids, glucose and waist circumference and completed a smartphone scanning assessment (MeThreeSixty^®^) to collect digital anthropometrics. Actual MetS classification and MetS severity (MetS_index_), a continuous estimate of MetS progression, were determined using MetS screening data. Then, least absolute shrinkage and selection operator regression was used to develop a new MetS_index_ prediction equation in a subset of participants (*n* 226), which was subsequently tested in the remaining participants (*n* 55), and MetS classification was predicted from the retained variables using logistic regression. The following equation was produced: *Smartphone-predicted MetS*_*index*_: −0·8880 + 0·1493(*medication use = 1; 0 = no medication use*) + 0·0089(*weight*) + 0·0079(*bust circumf.*) + 0·0140 (*thigh circumf.*) – 0·6247(*appendage-to-trunk circumf. index*), where *medication use* includes medications for hypertension, dyslipidaemia or hyperglycaemia. The newly developed MetS_index_ prediction model demonstrated equivalence with actual MetS_index_ and revealed acceptable agreement (R^2^:0·72; root mean squared error: 0·42; se of the estimate: 0·22) when evaluated in the testing sample (*n* 55), although proportional bias was observed (*P* < 0·001). Smartphone-predicted MetS classification demonstrated acceptable diagnostic performance with an accuracy of 92·7 % and an AUC of 0·89. Smartphone scanning applications can accurately assess MetS prevalence and severity, presenting new possibilities for health screening beyond clinical environments.

Metabolic syndrome (MetS), a condition characterised by the presence of several adiposity-related cardiometabolic abnormalities, is a prominent precursor of many chronic diseases^(1)^. In fact, MetS now mirrors obesity in its prevalence and recognition as a public health priority, with recent estimates suggesting that > 40 % of USA adults have MetS^(2)^. With the continuous rise in obesity, an ageing USA population more susceptible to cardiometabolic diseases^(3)^ and the increased rate of MetS amongst young adults^(4)^, it is unsurprising that integrating MetS screening techniques into routine care remains challenging for overwhelmed healthcare systems. Nevertheless, routine health screenings are critical for identifying those at elevated risk.

Screening for MetS typically involves evaluating individual risk factors such as abdominal obesity, hypertension, hyperglycaemia and dyslipidaemia. However, acquiring these diagnostic biomarkers is often challenged by cost, availability, technician dependence and access, particularly for those in rural and low socio-economic communities. Consequently, anthropometric measures are frequently used as primary indicators of cardiometabolic complications, given the relationship between MetS development and increasing adiposity. While BMI has historically been used to assess cardiometabolic health status due to its convenience, its oversimplified nature has led to a lack of clinical consensus. More recently, combining BMI with proxies of central adiposity, such as absolute and/or relative waist circumference, has been the preferred approach, as it may better represent fat distribution patterns indicative of cardiometabolic dysfunction^(5)^. However, these proxies often depend on access to trained personnel, measurement location and intra/interrater reliability. As a result, many desire more detailed body composition assessments, which have well-demonstrated associations with chronic disease^(6)^. Unfortunately, the most widely accepted methods are expensive and typically unavailable outside of research environments, and many consumer-level devices are inaccessible and often require patients to incur additional costs. Although most healthcare systems have begun addressing accessibility concerns through the rapid adoption of digital health services, most anthropometric methods cannot provide remote assessments without a technician present, and self-assessments are often inaccurate^(7)^. Given the limited clinical acceptance and feasibility of these approaches, it is essential to identify accessible, non-invasive, time- and cost-efficient anthropometric tools capable of evaluating MetS both remotely and at point-of-care.

A potential solution may lie in recent adaptations of 3-dimensional (3D) body scanning. Specifically, 3D scanning uses light emission and detection techniques to create a 3D avatar capable of automating hundreds of anthropometric measurements. While prior studies highlight the potential of this method as a MetS screening tool^(8,9)^, it remains limited by the same drawbacks as many previously described strategies. However, since most smartphones are equipped with the high-quality imaging and machine learning capabilities necessary for these assessments, 3D scanning procedures have now been optimised for smartphone applications. Although integration of this technique into smartphone applications represents a promising advancement in remote and automated cardiometabolic health screenings, the ability of smartphone-derived anthropometrics to effectively evaluate MetS remains unclear. Therefore, this study aimed to determine whether anthropometrics obtained from a smartphone application could be used to predict both MetS and its severity.

## Methods

### Participants

A total of 281 participants aged 18–65 years were prospectively recruited through a combination of convenience and snowball sampling methods (i.e. in-person and online word-of-mouth) and completed this cross-sectional evaluation. Participants were excluded if they were < 18 years or > 65 years; pregnant or breast-feeding/lactating. The age range for study eligibility was established based on the American Aging Association’s guidelines for clinically meaningful age groupings in the context of disease^(10)^. Moreover, this age group is at the highest risk for developing MetS, particularly among individuals in the younger subcategories^(4)^. Because the MetS severity (MetS_index_) equations used in this study are unavailable for Asian individuals^(11)^, Asian participants were also not included in this analysis. This study was conducted according to the guidelines laid down in the Declaration of Helsinki, and all procedures involving human subjects were approved by the University of Southern Mississippi Institutional Review Board (IRB#22-1012/23-0446). Written informed consent was obtained from all subjects.

### Procedures

Participants arrived at the laboratory for testing after an ≥ 8-h overnight fast from food, beverage and supplements/medications and after abstention from exercise for ≥ 24-h. Participants were instructed to wear tight form-fitting athletic clothing and to remove any external metal or accessories prior to testing. After sitting for a minimum of 5-min, systolic and diastolic blood pressure were collected using an automated digital blood pressure monitor. Afterwards, participants underwent several anthropometric evaluations including measurements of height, weight, waist circumference at the superior iliac crest using traditional tape measure and automated anthropometric assessments using a freely downloadable 3D smartphone application (MeThreeSixty^®^, Size Stream, Cary, NC) that provides users with both the images and anthropometric data free of charge (additional metrics provided via a $4·99 USD/month subscription at time of this study). Finally, capillary blood was collected to assess fasting blood glucose, HDL-cholesterol and TAG.

### Anthropometric smartphone application

Body composition, circumferences, surface areas and volumes were measured using a smartphone scanning application, and the procedures used to collect these measurements have been described in detail elsewhere^(12–16)^. Notably, the measurements produced by this smartphone application have well-demonstrated precision^(12,13,16,17)^ and have shown to agree with criterion methods^(12–15,17)^. Importantly, the mobile application used in this study has demonstrated acceptable agreement and test-retest reproducibility across multiple smartphone models^(13)^, including various Apple^®^ devices equipped with body scanning capabilities^(14)^. For these assessments, participants were required to wear only tight-form fitting athletic clothing and to tie their hair up so that it was not present below the shoulder line. After entering the participant’s descriptive information into the application, the smartphone (iPhone 14 Pro Max, Apple^®^, Cupertino, CA) was placed into a stationary tripod at a standardised height, and the smartphone’s orientation was confirmed by the application. Participants were then instructed to stand on top of a foot guide at a standardised distance from the smartphone. Once positioned, the application prompted participants to situate themselves into two poses: (1) the *A* pose, which required participants to face the smartphone, widen their feet and laterally raise their arms and (2) the *side* pose, which required participants to turn to the side, bring their feet together and place their hands to their sides. The smartphone’s front facing camera captured a single image during each pose. All scans were conducted in a designated area featuring a neutral-colored backdrop (grey) and no external light sources (e.g. window light) nor light at the participants back. Importantly, according to the manufacturer, raw image files are neither stored on the device nor uploaded to the cloud to protect user privacy.

### Blood biomarkers and metabolic syndrome assessments

The procedures for the collection of blood biomarkers and the determination of MetS and MetS_index_ have been published elsewhere^(18–22)^. In summary, ∼40 μl of capillary blood were collected via fingerstick, placed into to a single-use testing cassette and inserted into a validated capillary blood analyser^(23)^ (Cholestech LDX, Abbot, Abbott Park, IL) for the analysis of HDL-cholesterol (%CV: 3·3–4·9), TAG (%CV: 1·6–3·6) and fasting blood glucose (%CV: 4·5–6·2). Importantly, this capillary blood analyser does not report HDL-cholesterol for HDL-cholesterol < 15 mg/dl (*n* 3) nor TAG measurements > 650 mg/dl (*n* 3) and does not report TAG for TAG > 650 mg/dl nor TAG < 45 mg/dl (*n* 39). As such, HDL-cholesterol and TAG below these thresholds were recorded as 15 mg/dl and 45 mg/dl, respectively, and TAG > 650 mg/dl were recorded as 650 mg/dl. Because all participants with TAG > 650 mg/dl were classified as having MetS, irrespective of their HDL classification, HDL-cholesterol values for these participants were recorded as the median HDL-cholesterol of sex, race/ethnicity and MetS matched participants. Quality assurance tests were performed in compliance with the manufacturer’s standards.

MetS classification was determined using the ATP III criterion^(1)^, which includes possessing any three of the following five risk factors: (1) fasting blood glucose ≥ 100 mg/dl; (2) systolic blood pressure ≥ 130 mmHg or diastolic blood pressure ≥ 85 mmHg; (3) TAG ≥ 150 mg/dl; (4) waist circumference ≥ 88 cm for females and ≥ 102 cm for males and (5) HDL-cholesterol < 50 mg/dl for females and < 40 mg/dl for males. Participants that were currently being prescribed medications for the treatment of hypertension, hyperglycaemia and/or dyslipidaemia (*n* 17) were classified as meeting the criteria for the treated risk factor. The only other medications reported by participants with the potential to meaningfully influence cardiometabolic outcomes were muscle relaxants (*n* 3) and over-the-counter anti-inflammatory drugs (*n* 12). However, as part of the overnight fasting protocol, participants were instructed to abstain from taking these medications until after their study visit. Because these medications were used on an as-needed basis for personal or prescribed reasons and are not considered in the formal diagnostic criteria for MetS, they were not included in model development. Nonetheless, their prevalence is reported here to provide transparency and contextual clarity.

The primary outcome variable in the current study was MetS_index_, defined as an expansion of the simple dichotomous classification system (i.e. MetS positive or negative) into a continuous value whose magnitude represents the severity and progression of MetS. MetS_index_ scores were calculated from the aforementioned risk factors for each participant using the sex and race specific equations put forth by Gurka *et al.* ^(11)^. Interpreted as a z-score, positive and negative MetS_index_ values represent greater and lesser MetS severity and progression, respectively, and these values have shown to be associated with additional markers of cardiometabolic dysfunction^(11)^.

### Model development

A new MetS_index_ prediction equation was developed using least absolute shrinkage and selection operator (LASSO) regression procedures^(15)^ after employing the demographic and smartphone-derived anthropometric predictor variables listed hereafter. Demographic predictor variables included: age, height, weight, sex, race (White/Black), ethnicity (Hispanic/non-Hispanic), medication use (prescribed or not prescribed medication to treat hypertension, hyperglycaemia and/or dyslipidaemia) and smoking status. Anthropometric predictor variables produced by the smartphone application included: circumferences (cm) of the head, collar, neck, halter, shoulder, chest, bust (designated as *HingedBust* in the application), upper arms, biceps, forearms, wrists, thighs, knees, calves, ankles and vertical trunk; lengths (in cm) of the arms, outside legs and central trunk; surface areas (cm^2^) and volumes (cm^3^) of the whole-body, arms, legs and torso and appendage-to-trunk circumference index (ATI), defined as the sum of left and right upper arm, thigh, and calf circumferences divided by the stomach circumference^(24)^. Circumferences and lengths collected from the right and left sides were averaged to produce a single estimate, whereas right and left surface areas and volumes were summed. To evaluate whether smartphone-derived anthropometric measurements offered additional predictive value beyond demographic variables alone, we first developed a baseline model using only demographic predictors via LASSO regression. This was followed by the construction of a comprehensive model incorporating both demographic and smartphone anthropometric variables.

### Statistical analyses

Using a medium effect size (f^2^:0·25) and five predictor variables, power analyses for multiple linear regression revealed that fifty-seven participants would yield 80 % power at an *α* = 0·05. To create and assess a newly developed MetS_index_ prediction equation, a training dataset comprised of 80 % of the sample (*n* 226) and a testing dataset containing the remaining 20 % (*n* 55) were produced using random sampling techniques in R^(25)^. LASSO regression was then used to fit models in the training dataset using the *glmnet* package in R^(26)^. Importantly, LASSO regression works by identifying the predictor variables that decrease prediction error while simultaneously shrinking the coefficients of extraneous variables towards zero so that they are effectively omitted from the model^(27)^; ultimately producing the most parsimonious model that minimises multicollinearity and model overfitting. To determine the LASSO shrinkage technique, the best λ value was calculated using 10-fold cross-validation with the one se rule^(15)^.

After the MetS_index_ prediction model was developed in the training sample, the model was used to predict MetS_index_ in the testing sample. The performance of the smartphone-predicted MetS_index_ was evaluated against the actual MetS_index_ in the testing sample using paired *t* tests, equivalence tests, coefficients of determination (R^2^), Deming regression, Bland–Altman analyses, root mean squared error, se of the estimate, and concordance correlation coefficients. Because MetS_index_ is interpreted as a z-score, the equivalence regions were defined as ±0·34 to represent approximately one-third of a standard deviation, which have been used to assess z-score values in prior studies^(28)^. The agreement between smartphone-predicted MetS_index_ and the line-of-identity using Deming regression was determined if the 95 % confidence intervals for the intercept and slope contained the values 0 and 1, respectively. The 95 % limits of agreement and proportional biases were determined using Bland-Altman and linear regression techniques.

Additionally, the ability of the retained smartphone variables to correctly predict MetS classification was evaluated using binomial logistic regression with a cutoff point of 0·5 (MetS negative: < 0·5; MetS positive: ≥ 0·5). The true positive and true negative proportions of smartphone-predicted MetS were compared with the proportions determined by the actual diagnostic procedures using the receiver operating characteristic area under the curve (AUC), chi-square tests with corrections for continuity, R^2^_McFadden_, and sensitivity, specificity, accuracy, and positive (LR+) and negative (LR-) likelihood ratios. Acceptable accuracy of the smartphone-predicted MetS classification was defined as having a both an AUC ≥ 0·70 and having a summed sensitivity and specificity of ≥ 1·50^(29–31)^. Variance inflation factors were used to assess the multicollinearity of the final LASSO and logistic regression models (all Variance inflation factors ≤6·24). Statistical significance was accepted at *P* < 0·05.

## Results

### Metabolic syndrome severity

Descriptive characteristics of the participants in the combined, training, and testing samples are presented in Table 1. The initial model, which included only demographic predictors (intercept: −0·2925), retained age (*β* = 0·0028), medication use (*β* = 0·2692), height (*β* = −0·0113), and weight (*β* = 0·0234). However, this model performed worse overall (R^2^ = 0·63; 95 % limits of agreement = 0·82; AUC = 0·84; joint sensitivity and specificity = 1·48) compared with the full model that incorporated both demographic and smartphone-derived anthropometric variables (reported below). Notably, when LASSO regression was applied to the complete model, age and height were excluded, while the coefficients for medication use and weight were substantially reduced, indicating that the additional anthropometric features provided stronger predictive value.

**Table 1.** Descriptive characteristics of the combined, training and testing samples

	**Combined sample**			**Training sample**			**Testing sample**		
	***n***	**Mean**	**sd**	***n***	**Mean**	**sd**	***n***	**Mean**	**sd**
*n*	281			226			55		
Sex (F/M)	173/108			136/90			37/18		
Race (W/B)	170/111			133/93			37/18		
Ethnicity (H/NH)	17/264			9/217			8/47		
Metabolic syndrome	49			39			10		
Medication use	18			12			6		
Smoke	16			14			2		
Age (years)		24·5	8·7		24·6	8·8		24·3	8·3
Height (cm)		169·6	9·8		169·8	10·1		168·7	8·1
Weight (kg)		77·1	20·2		76·9	20·6		77·8	18·8
BMI (kg/m^2^)		26·7	5·9		26·5	5·9		27·3	6·1
Body fat (%)*		27·3	8·2		26·9	8·1		28·9	8·7
Fat mass (kg)*		21·7	11·4		21·3	11·4		23·3	11·6
Fat-free mass (kg)*		55·4	12·8		55·6	13·3		54·5	11·0
Metabolic syndrome severity score		–0·36	0·70		–0·33	0·71		–0·43	0·68
Smartphone metabolic syndrome severity score^†^	–				–			–0·28	0·43
Waist circumference (cm)*		88·5	14·4		88·1	14·3		90·2	14·6
Systolic blood pressure (mmHg)		117·2	13·4		117·6	13·4		115·7	13·5
Diastolic blood pressure (mmHg)		78·9	10·4		78·5	10·3		80·9	10·7
HDL-cholesterol (mg/dl)		48·4	13·9		48·0	14·3		50·4	12·1
TAG (mg/dl)		111·2	100·7		116·3	108·6		90·0	54·2
Fasting blood glucose (mg/dl)		89·7	8·1		89·8	7·8		89·4	9·1
Head (cm)		59·2	3·5		59·1	3·6		59·6	2·9
Collar (cm)		37·3	4·5		37·3	4·6		37·3	4·0
Neck (cm)		43·1	4·1		43·1	4·3		42·9	3·5
Halter (cm)		85·2	8·1		84·9	8·1		86·4	8·2
Shoulder (cm)		110·8	12·3		110·7	12·5		111·4	11·5
Chest (cm)		101·6	14·6		101·2	14·7		103·1	14·5
Bust (cm)		104·1	13·7		103·6	13·7		105·8	13·8
Upper arms (cm)		33·8	5·1		33·6	5·0		34·5	5·4
Biceps (cm)		31·6	5·1		31·5	5·0		32·2	5·5
Forearms (cm)		26·7	3·4		26·7	3·4		26·8	3·3
Wrists (cm)		16·4	1·7		16·4	1·7		16·5	1·6
Stomach (cm)		92·1	13·7		91·6	13·6		93·8	14·0
Waist (cm)		91·3	13·5		90·8	13·6		93·6	13·7
Hips (cm)		104·9	11·8		104·4	11·7		107·0	12·3
Thighs (cm)		59·9	7·7		59·5	7·4		61·4	8·7
Knees (cm)		38·3	3·9		38·1	3·8		39·0	4·4
Calves (cm)		38·0	3·9		37·9	3·9		38·6	4·0
Ankle (cm)		24·5	2·0		24·5	2·1		24·7	1·8
Arm length (cm)		58·6	4·2		58·6	4·4		58·3	3·2
Outside leg length (cm)		103·3	6·4		103·4	6·7		103·0	4·5
CTL (cm)		154·5	13·2		154·4	13·6		155·0	11·7
VTC (cm)		162·4	14·9		162·2	15·3		163·3	13·3
Surface area (cm^2^)		18·201	2·716		18·156	2·764		18·385	2·524
Arm surface area (cm^2^)		3·280	564		3·280	575		3·278	521
Leg surface area (cm^2^)		8·804	1·211		8·778	1·227		8·914	1·149
Torso surface area (cm^2^)		6·120	1·056		6·102	1·078		6·191	968
Body volume (cm^3^)		75·494	21·854		74·981	21·969		77·605	21·444
Arm volume (cm^3^)		8·134	2·632		8·109	2·623		8·236	2·693
Leg volume (cm^3^)		19·629	5·036		19·430	4·880		20·449	5·606
Torso volume (cm^3^)		47·661	14·933		47·373	15·190		48·841	13·892
ATI		2·88	0·19		2·87	0·19		2·88	0·15

Data are presented as *n* or mean (standard deviation).

All body circumferences from the right and left sides were averaged to produce a single estimate. Appendicular surface areas and volumes were calculated as the sum of the right and left sides. For ATI, all variables were collected using smartphone-derived measurements. Medication use was defined as being prescribed medication to treat hypertension, hyperglycaemia and/or dyslipidaemia. Smoking was defined as currently smoking or vaping.

F, female; M, male; W, White; B, Black; H, Hispanic; NH, non-Hispanic; CTL, centre trunk length; VTC, vertical trunk circumference; ATI, appendage-to-trunk circumference index.

*Estimates produced using smartphone application; ^†^estimates produced using the newly developed smartphone prediction model.

The coefficients of the retained variables using LASSO regression in the complete model are presented in Table 2. Retained variables included medication use, weight, bust circumference, thigh circumference, and ATI, which produced the following equation:

**Table 2.** LASSO regression model coefficients predicting metabolic syndrome severity from smartphone-derived anthropometrics

**Predictor variables**	**Metabolic syndrome severity prediction coefficients**
Intercept	–0·8880
Medication use	0·1493
Weight (kg)	0·0089
Bust (cm)	0·0079
Thighs (cm)	0·0140
ATI	–0·6247

LASSO, least absolute shrinkage and selection operator; ATI, appendage-to-trunk circumference index.

All data are presented as the unstandardised coefficients for each variable within the corresponding body composition prediction model. The coefficients of all other predictor variables were shrunk to ‘0’ and are therefore not included in the final model equations nor the table.

‘Medication use’ was defined as 1 = prescribed medication to treat hypertension, hyperglycaemia and/or dyslipidaemia or 0 = no prescribed these medications. For ATI, all variables were collected using smartphone-derived measurements and was defined as the sum of left and right upper arm, thigh and calf circumferences divided by the stomach circumference.

*Smartphone-predicted MetS*_*index*_: −0·8880 + 0·1493(*medication use = 1; 0* = *no medication use*) + 0·0089(*weight*) + 0·0079(*bust circumf.*) + 0·0140(*thigh circumf.*) – 0·6247(*ATI*).

**Figure 1.**
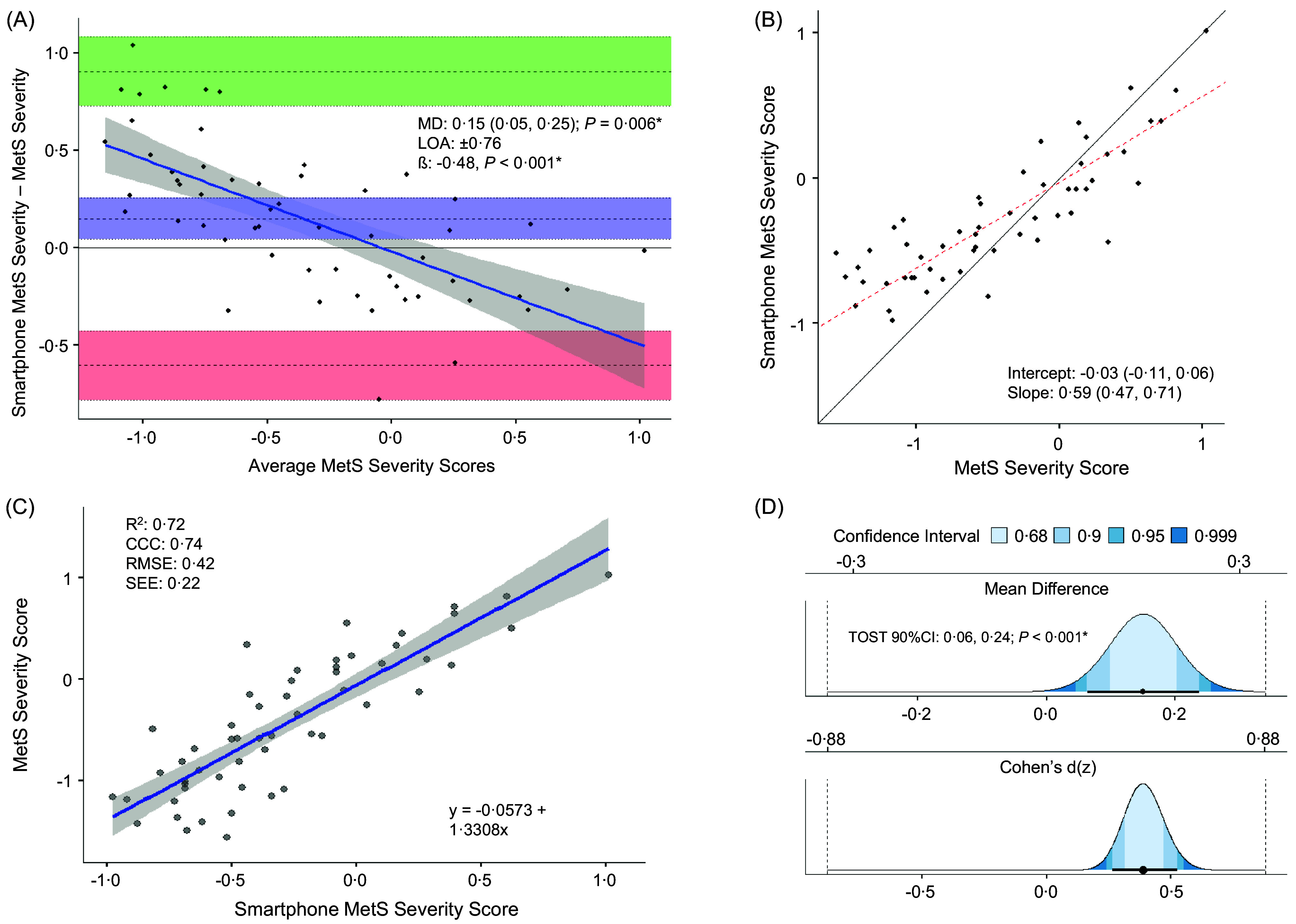
(a)–(d) Bland–Altman (a), Deming regression (b), simple regression (c) and equivalence plots demonstrating the agreement between smartphone-predicted MetS_index_ and the actual MetS_index_ in the testing sample (*n* 55). For the Bland–Altman plots (a), the upper and lower dashed lines represent the 95 % LOA, the middle-dashed line represents the MD between the smartphone-predicted MetS_index_ and the actual MetS_index_ and the solid blue line and its corresponding shaded area represents the regression line and its 95 % CI, respectively. For the Demming regression plots (b), the solid black line represents the line of identity and the red dashed line represents the regression line. For the simple regression plot (c), the solid blue line and its corresponding shaded area represents the regression line and its 95 % CI, respectively. For the equivalence plots, the average MD (top) and effect size MD (bottom) are presented, where the blue shaded regions represent the TOST CI displayed in the CI legend, the black circles and intersecting horizontal lines represent the MD and the TOST 90 % CI, respectively, and the vertical dashed lines indicate the equivalence regions. *β*, proportional bias coefficient; CCC, concordance correlation coefficient; LOA, 95 % limits of agreement; MD, mean difference calculated as the smartphone-predicted MetS_index_ minus the actual MetS_index_.; MetS_index_, metabolic syndrome (MetS) severity score; R^2^, coefficient of determination; RMSE, root mean square error; TOST, values from the TOSTER package in R. * statistically significant at *P* < 0·050.

The equation presents unstandardised coefficients for the predictors retained in the final model. All other predictor variables, as detailed in the preceding sections, had their coefficients shrunk to 0, effectively excluding them from the model. Figure 1(a)–(d) illustrates the agreement between the newly developed smartphone MetS_index_ prediction equation and the actual MetS_index_ in the testing sample. Paired *t* tests revealed significant differences between smartphone-predicted and actual MetS_index_ (*P* = 0·006; Figure 1(a)), though smartphone-predicted MetS_index_ demonstrated equivalence with actual MetS_index_ (Figure 1(d)). Online Supplemental Table 1 presents the prevalence and risk factors of MetS across MetS_index_ groups, as defined by these equivalence bounds, to further support the results of our equivalence testing procedures. R^2^, concordance correlation coefficient, root mean squared error and se of the estimate values (Figure 1(c)) revealed good agreement between methods, and 95 % limits of agreement were moderate. Proportional biases (*P* < 0·001; Figure 1(a)) and differences in the slope (but not intercept) from the line-of-identity were observed (Figure 1(b)).

### Metabolic syndrome classification

**Figure 2.**
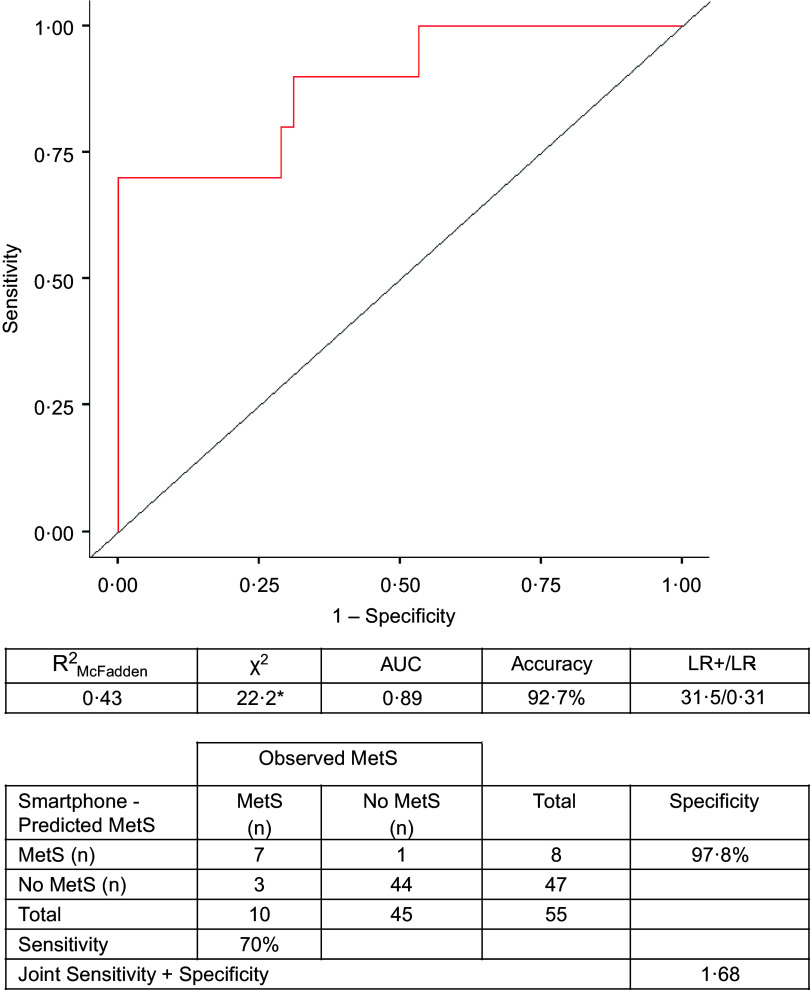
An ROC curve and corresponding AUC demonstrating the ability of the predictor variables retained during LASSO regression to predict conventional MetS classification relative to actual MetS status in the testing sample (*n* 55). Positive and negative MetS cases are presented for both smartphone-predicted and actual MetS, as well as the sensitivity, specificity, and accuracy of smartphone-predicted MetS status. R^2^_McFadden_, χ^2^, and LR+ and LR- are also tabulated. LASSO, least absolute shrinkage and selection operator; LR+, positive likelihood ratio; LR-, negative likelihood ratio; MetS, metabolic syndrome; R^2^_McFadden_, McFadden pseudo coefficient of determination; ROC, receiver-operating characteristic. * statistically significant at *P* < 0·050.

The receiver operating characteristic plots and associated contingency tables of Figure 2 demonstrate the ability of the retained smartphone variables to accurately diagnose MetS compared with the traditional diagnostic standards. The true prevalence of MetS was 18·2 %, whereas the apparent prevalence of smartphone-predicted MetS was 14·5 %. *χ* ^2^ tests for the overall model were significant (*P* < 0·001), and smartphone-predicted MetS had a diagnostic accuracy of 92·7 %, with sensitivity (70 %), specificity (97·8 %; joint sensitivity + specificity: 1·68), and LR+ (31·5) and LR− (0·31) indicating acceptable diagnostic performance. Moreover, the model AUC (0·89) and R^2^_McFadden_ (0·43) revealed excellent model performance.

## Discussion

After developing a MetS_index_ prediction model using LASSO regression and cross-validating this model in an independent subset of participants, our evaluations revealed that smartphone-acquired anthropometrics can provide accurate estimates of MetS severity and progression. While the ability to predict a continuous MetS_index_ score from a smartphone application may improve diagnostic flexibility and better inform clinicians of MetS progression, the absence of diagnostic cut-offs may complicate clinical decision-making and limit the implementation of this technique at scale. Therefore, we also assessed whether smartphone-derived anthropometrics could accurately predict MetS classification, and our model demonstrated good-to-excellent ability to identify MetS in accordance with traditional diagnostic standards. These findings affirm that both MetS and its severity can be accurately determined using a smartphone application.

The ability of 3D body scanners to deliver accurate estimates of conventional anthropometrics is well established^(32)^. However, ongoing advancements in artificial intelligence and digital imaging now offer new opportunities to leverage the predictive power of these automated anthropometrics to produce more clinically significant outcomes. For example, 3D scanners have accurately predicted measurements such as appendicular lean mass^(15)^ and MetS^(9)^ based on the established relationship between key anthropometrics and both muscularity^(33)^ and chronic disease^(34)^. In fact, prior studies have demonstrated that body volumes obtained from 3D scanners predict MetS more accurately than traditional anthropometric assessments^(5)^, which is likely attributed to the advanced landmarking procedures that can eliminate human error while simultaneously automating more elaborate measurements. However, given the existing limitations of 3D scanning, manufacturers have begun to couple 3D scanners with smartphone equivalents, which have demonstrated a similar ability to predict MetS^(5)^.

Our findings align with previous studies that employed both 3D^(5,9)^ and smartphone^(5)^ scanning techniques for predicting MetS classification. However, to our knowledge, only one study has assessed whether a smartphone application can predict the prevalence and severity of MetS^(5)^. Although the predictive power of our smartphone scanning model for detecting binary MetS status was nearly identical to other methods^(5,9)^, our model exhibited markedly greater accuracy in predicting MetS_index_^(5)^. While the improved performance of our model could be due to the technological differences between applications, it could also result from variations in the strength of the predictor variables. For example, other applications use body volume estimates to predict MetS, which have shown comparable performance to traditional circumferences collected from similar regions^(8)^. However, previous studies have shown that the prediction of MetS^(9)^ and MetS risk factors^(35)^ improves most when using automated circumferences. Since body circumferences appear to be stronger predictors of binary MetS status, and MetS_index_ is simply an extension of this status, the systematic selection and omission of body circumferences and volumes, respectively, likely accounts for the performance differences between applications.

Importantly, since waist circumference is a risk factor for MetS and is included in MetS_index_, we chose to exclude closely related raw circumferences as predictor variables. This allowed us to avoid potential biases and addressed the challenges of predicting MetS_index_ from abdominal circumferences in Black adults^(21)^. Despite excluding these variables, we still observed acceptable model performance using only circumferences of the bust, thighs and ATI. Recent investigations using artificial intelligence to predict diabetes and hypertension from biometric data have shown that thigh circumference enhances model specificity more than BMI; likely because it can better discern young adults with greater muscularity from those with higher body fat^(36)^. While it is reasonable to assume that bust circumference was included as a marker of upper body adiposity in the absence of raw abdominal measurements, bust circumference may also uniquely contribute to MetS. Exploratory analyses of the present dataset revealed that bust, but not waist circumference (*r*: –0·09, 0·00; *P* > 0·050), was significantly associated with systolic blood pressure (*r*: 0·30, *P* = 0·029) and HDL-cholesterol (*r*: –0·27, *P* = 0·047) after adjusting for both age and BMI. Interestingly, HDL-cholesterol is inversely associated with estradiol^(37)^, which contributes to fat accumulation in breast tissue^(38)^. Greater fat accumulation could stimulate aromatase activity, and elevated conversion of androgens-to-estrogens may increase bust size^(39)^ whilst exerting vasoconstrictive responses that increase systolic blood pressure^(40)^. Despite the historical reliance on abdominal measurements to assess cardiometabolic abnormalities, our findings suggest that individual components of MetS could be more effectively captured using alternative anthropometrics, and mobile scanning presents an emerging opportunity to automate anthropometrics that have traditionally been difficult to obtain.

There are a few limitations that warrant discussion. While our sample was diverse, we were unable to evaluate Asian adults, as MetS_index_ is currently unavailable for this group. The average age of our sample was also relatively young. However, the prevalence of MetS in our sample matches the national prevalence for this age range and reflects the age group experiencing the most rapid increase in MetS^(4)^. Although age was included as a continuous predictor in the initial LASSO regression model, it was not retained in the final model, suggesting that it did not contribute additional predictive value beyond the selected variables. Similarly, sex was included as a predictor but was excluded during model selection. While future models evaluating the performance of smartphone-derived anthropometrics for predicting MetS_Index_ within sex-specific subgroups may be warranted, the sample sizes in our study were insufficient to support adequately powered stratified analyses and may have introduced bias into subgroup-specific models. Future studies seeking to improve these models should consider employing age- and sex-stratified models. The use of prescription medication to treat specific MetS risk factors may have lowered MetS_index_ for these participants. However, we accounted for this during LASSO, and medication use was retained as a variable in the final model. Because diastolic blood pressure is not included in MetS_index_, unique hypertensive MetS phenotypes may have been overlooked^(21)^. The prediction model in our study exhibited significant proportional biases and differences from the line-of-identity, which, in the context of chronic disease diagnosis, may lead to underestimation of risk among high-risk individuals. However, such biases, particularly underestimation, are commonly reported in studies validating novel anthropometric and body composition methods. Despite this, our model demonstrated excellent classification accuracy for MetS, supporting its potential utility in risk stratification. Still, users and practitioners should employ caution when using this technique. Because the mobile application used in our study has well-established test-retest reliability^(12,13,16,17)^, we did not collect duplicate measures of the smartphone-derived anthropometric variables and therefore cannot report test-retest reproducibility for these features (thigh, ATI and bust circumference) within the context of this study. However, previous studies have demonstrated the reproducibility of the thigh and chest circumferences produced by this mobile application, as well as the circumferences used to calculate ATI^(14,17)^. Although, to our knowledge, no studies have specifically evaluated the test-retest reproducibility of bust measurements using this mobile application, unpublished pilot data from our laboratory in an independent sample of female participants demonstrated acceptable agreement between smartphone-derived and tape-measured bust measurements (R² = 0·82; mean difference = 3·07 cm). Additionally, other studies have reported acceptable agreement between smartphone-derived and MRI-based breast volume estimates^(41)^. Together, these findings support the reliability and/or validity of the anthropometric variables retained in our prediction model. Finally, our findings are specific to the application used in our study.

In conclusion, smartphone scanning applications can accurately assess MetS prevalence and severity, supporting its use in remote settings. While longitudinal studies are needed to determine its effectiveness in monitoring changes, our findings highlight the predictive power of automated anthropometrics and present new possibilities for health screening beyond clinical environments.

## Supporting information

Brandner et al. supplementary material Brandner et al. supplementary material

## Supplementary material

For supplementary material/s referred to in this article, please visit https://doi.org/10.1017/S000711452510576X.
