# Supplementary material for: Development of a metabolic syndrome prediction model using smartphone-derived digital anthropometry
Source: Br J Nutr. 2025 Nov 21;135(2):232–40. doi: 10.1017/S000711452510576X (PMC12825942; doi:10.1017/S000711452510576X)
Supplement: Brandner et al. supplementary material [file S000711452510576Xsup001.pdf]

Supplemental Table 1. Metabolic syndrome prevalence and risk factors across severity groups defined by equivalence bounds

	MetS Severity Score < - 0.34	MetS Severity Score -0.34 to 0.34	MetS Severity Score > 0.34
	**N (%)**	**N (%)**	**N (%)**
No MetS	166 (99.4%)	54 (73.0%)	12 (29.3%)
MetS	1 (0.6%)	20 (27.0%)	28 (70.0%)
	**Mean ± SD**	**Mean ± SD**	**Mean ± SD**
WC (cm)	81.2 ± 8.6	94.0 ± 10.5 [a]	108.8 ± 15.8 [a, b]
SBP (mmHg)	114.6 ± 12.5	120.6 ± 13.5 [a]	121.7 ± 14.7 [a]
DBP (mmHg)	77.3 ± 10.0	79.3 ± 8.2	85.0 ± 13.3 [a, b]
HDL-C (mg/dL)	54.1 ± 11.9	42.5 ± 12.3 [a]	35.6 ± 10.7 [a, b]
TRG (mg/dL)	81.2 ± 54.2	117.4 ± 95.8 [a]	224.8 ± 161.3 [a, b]
FBG (mg/dL)	87.2 ± 7.1	92.0 ± 6.9 [a]	96.1 ± 9.0 [a, b]

Data are presented as N (percent of the column total) or as mean ± standard deviation

χ^2 test of independence: $\chi^2 = 114.0$; $p < 0.001$; omnibus one-way ANOVA: all risk factors resulted in $p \leq 0.003$

[a] significantly different from the < - 0.34 MetS severity score group at p < 0.050 using Tukey post-hoc tests; [b] significantly different from the - 0.34 to 0.34 MetS severity score group at p < 0.050 Tukey post-hoc tests; [c] significantly different from the > 0.34 to 0.34 MetS severity score group at p < 0.050 using Tukey post-hoc tests.

WC: waist circumference; SBP: systolic blood pressure; DBP: diastolic blood pressure; HDL-C: high-density lipoprotein cholesterol; TRG: triglycerides; FBG: fasting blood glucose
